# Does time to loss to follow-up differ among adult tuberculosis patients initiated on tuberculosis treatment and care between general hospital and health centers? A retrospective cohort study

**DOI:** 10.1186/s41182-020-00198-8

**Published:** 2020-02-18

**Authors:** Tamrat Shaweno, Masrie Getnet, Chaltu Fikru

**Affiliations:** Department of Epidemiology, Faculty of Public Health, Jimma University Institute of Health, Jimma, Ethiopia

**Keywords:** Ethiopia, Loss to follow-up, Time to loss to follow-up, Tuberculosis

## Abstract

**Background:**

Patients’ loss to follow-up (LTFU) from tuberculosis treatment and care is a growing worry in Ethiopia. But, available information is inadequate in assessing the time to tuberculosis patient loss to follow-up difference between health centers and a general hospital in Ethiopia. We aimed to assess time to LTFU difference between health centers and a general hospital in rural Ethiopia.

**Methods:**

We conducted a retrospective cohort study from September 2008 to August 2015 and collected data from September 1 to October 02, 2016. A total of 1341 TB patients with known treatment outcomes were included into the study. Log rank test was used to compare the difference in time to TB patient loss to follow-up between health centers and a general hospital, whereas Cox proportional hazard model was used to assess factors associated with time to loss to follow-up in both settings.

**Results:**

We reviewed a total of 1341 patient records, and the overall follow-up time was 3074.7 and 3974 person months of observation (PMOs) for TB patients followed at health centers and a general hospital, respectively. The incidence of loss to follow-up rate was 27.3 per 1000 PMOs and 9.6 per 1000 PMOs, at health centers and a general hospital, respectively. From the overall loss to follow-ups that occurred, 55 (65.5%) and 33 (86.8%) of LTFUs occurred during the intensive phase and grew to 78 (92.9%) and 38 (100%) at health center and a general hospital, respectively, at the end of 6-month observation period. Older age (AOR = 1.7, 95%CI, 1.2–2.5, *P* < 0.001), being a rural resident (AHR = 2.7, 95%CI, 1.6–4.6), HIV reactive (AHR = 2.2, 95%CI, 1.5–3.2), following treatment and care in health center (AHR = 3.38, 95%CI, 2.06–5.53), and living at more than 10 km away from the health facility (AHR = 3.4, 95%CI, 2.1–5.7) were predictors for time to loss to follow-up among TB patients on treatment and care.

**Conclusion:**

Time to TB patient loss to follow-up between health centers and a general hospital was significant. Loss to follow-up was high in patients with older age, rural residence, sero positive for HIV, living further from the health facilities, and following treatment and care at health centers. Strengthening the DOTs program with special emphasis on health centers is highly recommended.

## Introduction

Although, highly efficacious treatment was available for decades, tuberculosis (TB) remains a major global public health problem [1]. In 2017, 10.5 million new cases and 1.5 million deaths were reported due to TB [1, 2]. Sub-Saharan Africa (SSA) accounts for the lion’s share of TB deaths, TB–HIV co-infections, and TB–HIV deaths. One underlying reason is the relatively low coverage and quality of TB prevention, treatment, and care programs in Africa [3, 4].

Ethiopia is among the 22 high burden TB countries and ranked tenth globally and fourth in Africa [2, 5]. Although there are remarkable achievements in the reduction of TB mortality from 2004 to 2014, there were an estimated 30,000 mortalities per annum and more than 80 TB associated deaths every day in Ethiopia during the same reporting period due to uncontrolled loss to follow-up [6, 7]. Loss to follow-up is believed to be the most significant indicator for a high burden of multidrug-resistant tuberculosis (MDR-TB). According to a recent systematic-review report, the pooled estimate of MDR-TB among new and previously treated cases attributable to loss to follow-up was 2% and 15%, respectively [8]. Even though about 85% of TB cases in Ethiopia occur in rural settings, the timing in loss to follow-up of TB patients following treatment and care in health facilities differing by their level of tiers remains undescribed [9]. Prior findings on TB patient loss to follow-up in Ethiopia did not consider the effect of health facilities differing in their level of service comprehensiveness on TB patients’ loss to follow-up, so it was difficult to inform the decision makers where and when to emphasize on TB patient monitoring and follow-up [10–12].

According to WHO, the directly observed treatment and short-course (DOTS) strategy is recommended to upgrade TB prevention and control [12] and Ethiopia has implemented this since 1991 [13, 14]. Consequently, about 92% of public hospitals and health centers in Ethiopia provide DOTS [15]. Loss to follow-up of TB patients is troubled with problems, primarily because of MDR-TB [16–18]. Non-adherence to complete treatment poses a substantial public health menace through disease recurrence, amplified transmission, and development of drug resistance [19, 20]. Besides the widespread expansion of DOTS services and the enormous participation of health extension workers (HEWs) in TB prevention and control activities, the patients still are failing to adhere to complete their treatment [21]. According to WHO 2017, substantial TB cases failed following some treatments and countless were relapsing and subjected to retreatment after completion of treatment [22]. The underlying reason for this was partly due to loss to follow-up of TB patients. Loss to follow-up from treatment and care is defined as a TB patient who did not start treatment or whose treatment was interrupted for two consecutive months or more [23]. In addition, the rate of loss to follow-up across different tiers of health systems is becoming recognizable currently. Understanding the time when TB patients’ loss to follow-up and the factors that predict the loss to follow-up are the bridges for developing time relevant intervention approaches. This study aimed at determining whether time to loss to follow-up differs between health centers and a general hospital; and associated factors among TB patients in Ethiopian rural health facilities.

## Methods

### Setting, design, and population

This is a retrospective study on TB patients registered for TB treatment from 21/09/2008 to 11/08/2015 in Sheka Zone, Ethiopia, which is located at 687 km away from the national capital, Addis Ababa. Sheka Zone is divided into three administrative districts, which are further divided into 66 kebeles, the smallest geographical administrative units in Ethiopia. Among the 66 kebeles, all six urban kebeles and 22 rural kebeles were located within 10 km distance from the nearest health facility (it applies for those only seven health centers and one general hospital included into this study) [24]. There are 14 health centers and one general hospital in the study catchment; even though during the study period only seven had health centers and one general hospital had functional laboratories for TB sputum smear microscopy and none had access to X-ray or culture facilities, only 7 health facilities were included into this study. Diagnosing and treating TB in Ethiopia is based on the Ethiopia’s national TB treatment guidelines [25]. Accordingly, a tuberculosis patient was classified as having smear-positive pulmonary TB (PTB+) if the patient has at least two initial sputum smear examinations positive for AFB by direct microscopy, or a patient has one initial smear examination positive for AFB by direct microscopy and culture positive, or a patient has one initial smear examination positive for AFB by direct microscope and radiographic abnormalities consistent with active TB as determined by a clinician. Similarly, a patient having symptoms suggestive of TB with at least three initial smear examinations negative for AFB by direct microscopy, and no response to a course of broad-spectrum antibiotics, and again three negative smear examinations by direct microscopy, and radiological abnormalities consistent with pulmonary tuberculosis, and decision by a clinician to treat with a full course of anti-tuberculosis or patient whose diagnosis is based on culture positive for *M*. *tuberculosis* but three initial smear examinations negative by direct microscopy was considered as smear-negative pulmonary TB (PTB−). Patients categorized as having extra-pulmonary tuberculosis (EPTB) were patients having TB in organs other than the lungs, proven by one culture-positive specimen from an extra-pulmonary site or histo-pathological evidence from a biopsy, or TB based on strong clinical evidence consistent with active EPTB and the decision by a physician to treat with a full course of anti-TB therapy. Patients with negative sputum smears who fail to respond to treatment with broad-spectrum antibiotics are considered to have smear-negative pulmonary TB [25, 26], although the diagnosis of smear-negative and extra-pulmonary cases also incorporates clinical judgment. Patients receive daily rifampicin, pyrazinamide, isoniazid, and ethambutol for 2 months (initial phase) followed by daily rifampicin and isoniazid for 4 months or more (continuation phase). Patients diagnosed with TB are registered in a TB unit register for DOTS at their presenting health facility; information on name, kebele of residence, age, sex, weight, type of facility (hospital or health center), sputum smear result, TB type, TB patient treatment category, HIV sero status, use of chemo prophylactic therapy (cotrimoxazole), anti-retroviral treatment (ART) status, anti-tuberculosis treatment regimen, treatment outcome, and dates of treatment initiation and treatment outcome are recorded.

All TB patients treated for TB according to the national TB treatment guidelines [25] from 2008 to 2015 and registered in the TB unit register log book with known treatment outcomes were included into the study. On the contrary, the patients with undocumented treatment outcome were excluded from the study. A total of 1504 patients had been registered in seven public health centers and one general hospital of which 161 patients were excluded from the study due to not documented status of outcomes and transferred out to other health facilities.

### Sample size determination and sampling techniques

The sample size was calculated using the sample size calculations for survival analysis [27]. In this calculation, the number of event (NE) was primarily calculated followed by probability of the event (Pr(event). Accordingly, the number of events was calculated using the following formula.

Number of events (NE) = $\frac{\left(\mathrm{Z}\frac{\alpha }{2}+\mathrm{Z}\upbeta \right)2}{\uppi 1\uppi 2\ \left(\log HR\right)2}=342$, where $\mathrm{Z}\frac{\alpha }{2}$ =1.96, Zβ =0.842, π_1_ and π_2_ are the proportions to be allocated to groups 1 and 2. For equal allocation, π_1_ = π_2_ = ½. Since similar studies that detect the survival time difference between health center and hospital were not available during the time this study was conducted, hazard ratio of 0.5 was considered. The probability of events was also calculated using the exponential survivorship function, S (*t*) = exp. {−λt}. Since, this study was a 7-year retrospective cohort (2008–2015); S (*t*) = exp. {− 0.7}. Accordingly, the probability of the event for group 1 was exp. {− 0.7}, which equals 0.5. Similarly, the probability of the event for group 2 was calculated using exp. {− 0.7*0.5}, since we used a hazard ratio of 0.5, and which becomes 0.7. Thus, the probability of the event becomes 1 − (0.4 + 0.7)/2 = 0.4. Thus, the final sample size was calculated using the following formula.

Sample size (*n*) = ($\frac{number\ of\ events\ }{Probablity\ of\ events}\Big)$ = 855, approximately 428 for each groups. We have also applied Interim Analysis Adjustment (IAA) to make an adjustment for data incompleteness. Accordingly, deciding to use a Pocock boundary of 1.2 at power of 80 for three interim analyses plus final, we have made an adjustment as follows.

Number of adjusted events = 342*1.2 = 411 and number of adjusted sample size becomes 1028 (411/0.4). After adjusting for 10% loss to follow-up rate (1028/0.9), the final sample size becomes 1143, approximately 572 for each group. But, from the health center tuberculosis patient registration log book, we found a total of 614 eligible tuberculosis patients and 727 eligible tuberculosis patients from the general hospital tuberculosis patient follow-up log book. Accordingly, 614 patients from the health center and 727 from the general hospital were included into this study. Therefore, a total of 1341 tuberculosis patients enrolled for treatment and care from 21/09/2008 to 11/08/2015 in all public health facilities were included into the study. Since we included all available and eligible records, we did not use any sampling method.

### Study variables and measurement

The dependent variable of interest was time to loss to follow-up, which was defined as patients who took treatment for at least 1 month and discontinue treatment for more than consecutive 8 weeks [24]. Records of patients registered to TB follow-up between 21/09/2008 and 11/08/2015 were reviewed to identify those who failed to keep scheduled appointments for more than 2 months. A list of these “loss to follow-up” patients was generated from medical register by observing the last treatment date. Death was considered as the death of TB patients on of follow-up in the reporting period due to any cause. A patient is cured who was initially smear-positive and who was smear-negative in the last month of treatment and on at least one previous occasion. Similarly, a patient who completed treatment but for whom smear results are not available at 7 months or 1 month prior to the completion of treatment is declared as treatment completed. Furthermore, if for whatever reason after 7 months of treatment the final sputum examination cannot be done and the sputum result at 5th month was negative or not done, the patient is declared treatment completed.

Treatment failure is declared when a patient whose sputum smear or culture is positive at 5 months or later during treatment or patients found to harbor a multidrug-resistant (MDR) strain at any point of time during the treatment, whether they are smear-negative or smear-positive. This definition applies to pulmonary smear-positive and smear-negative patients and to patients with extra-pulmonary disease. Transfer out is the official transferring of the patient to another TB clinic within or outside a catchment area [28, 29]. Independent variables included in this study were as follows: age of the TB patient, sex, distance from the nearest health facility (with in 10 km or more than 10 km distance), and residency (rural or urban) among the demographic information’s recorded for each patient. Baseline TB treatment category (new, relapse, failure, transferred in), HIV sero status (reactive, non-reactive), anti-retroviral treatment status (linked to ART, not linked to ART) status, anti-tuberculosis treatment regimen, treatment outcome, and dates of treatment initiation and treatment outcome are recorded. Data extraction tools were developed from WHO, national treatment guideline for TB treatment, and similar other studies [4, 6, 13, 24, 25, 28, 29]. To assure the data quality, the data were collected through structured data extraction formats developed from standard sources and data were collected by trained diploma nurses. Data collection process was supervised by two trained nurses holding Bachelor’s degree to assure the completeness of data.

### Data processing and analysis

The completed questionnaire was cleaned, checked for completeness, coded, and edited before data entry. The data were entered to EpiData 3.1 and exported to SPSS 23 for analysis. Descriptive statistics including frequency, percentage, median, and standard deviation of the study variables were computed. Cox proportional hazard model was applied to estimate the effect of risk factors. Variables with the 푝 < 0.25 during the bivariate analysis were included into a multiple cox model. A 95% CI and 푝 value of < 0.05 for multiple cox model were considered statistically significant. Loss to follow-up rates were calculated by summing the number of patients’ who experienced the event loss to follow-up during a particular period of time divided by the total number of years of follow-up during this period. The difference in proportions of patients surviving at some time (*t*) was described using Kaplan-Meier survival plot. The proportional hazard assumption was checked by using log-log survival curves based on Schoenfeld residuals. We calculated incidence density for loss to follow-up using person months (PMOs) of contribution to the cohort. Patients who completed treatment or active at the end of observation period were considered censored. Kaplan-Meier method and log rank test were used to estimate survival probability and statistical significance for categorical covariates.

## Result

### Description of the cohort

A total of 1341 eligible tuberculosis patients were included into this study. The distribution of the study participants by sex was approximately the same in health center and general hospital. Accordingly, 356 (58%) from health centers and 431 (59.3%) from the general hospital were males by sex. With regard to residence of the study participants, 184 (30%) from health centers and nearly all 720 (99.0%) from general hospital were residents of a rural community. Concerning patients’ access to health facility, 429 (69.9%) of patients taking care in health centers and 715 (98.3%) on follow-up and care at general hospital had been residing within a 10-km distance. The baseline body weight was normally distributed for both TB patients receiving treatment care in both health centers and general hospital. Accordingly, the mean body weight was 48.7 kg with SD of 7.8 kg and 50.2 kg with SD of 7.8 kg for TB patients receiving treatment and care at health centers and hospital, respectively.

The smear negativity is almost the same for both patients diagnosed at health centers and general hospital. More than 85% of the study participants in both health centers and general hospital were new TB patients. From a total of TB patients who were tested for HIV, 90 (14.7%) and 144 (19.8%) were reactive for HIV at health centers and general hospital, respectively (Table 1).

**Table 1:** Selected socio-demographic and clinical characteristics of 1341 TB patients enrolled into TB treatment and care at public health facilities, rural Ethiopia, during 2008–2015

Characteristics	Categories	Health centers	General hospital	Total
		*n* (%)	*n* (%)	*n* (%)
Age (in years) (*n* = 1341)	Continuous			
Sex (*n* = 1341)	Male	356 (58)	431 (59.3)	787 (58.7)
	Female	258 (42)	296 (40.7)	554 (41.3)
Residence (*n* = 1340)	Urban	184 (30)	720 (99.0)	904 (67.4)
	Rural	430 (70)	6 (0.8)	436 (32.6)
Baseline weight (*n* = 1341)	Continuous			
Patient category (*n* = 1341)	New	544 (88.6)	621 (85.4)	1162 (86.7)
	Relapse	14 (2.3)	19 (2.6)	35 (2.6)
	Failure	1 (0.2)	2 (0.3)	3 (0.07)
	Transferred in	55 (9.0)	85 (11.7)	141 (10.5)
Distance (*n* = 1340)	≤ 10 km	429 (69.9)	715 (98.3)	1144 (85.3)
	> 10 km	185 (30.1)	12 (1.7)	196 (14.6)
Drug intensive (*n* = 1341)	RHZE	606 (98.7)	724 (99.6)	1330 (99.2)
	RHZ	8 (1.3)	3 (0.4)	11 (0.8)
Drug continuous (*n* = 1322)	EH	232 (37.8)	225 (30.9)	461 (34.9)
	EH and RH	14 (2.3)	21 (2.9)	45 (3.4)
	RH	354 (57.7)	476 (65.5)	816 (61.7)
CPT provision at enrolment (*n* = 230)	Yes	63 (84.0)	138 (89.1)	201 (87.4)
	No	12 (16.0)	17 (10.9)	29 (12.6)
TB type (*n* = 1341)	P/pose	236 (38.4)	227 (31.2)	463 (34.5)
	P/negative	232 (37.8)	278 (38.2)	510 (38.0)
	EP	146 (23.8)	222 (30.5)	368 (27.4)
Treatment phase (*n* = 1341)	Intensive	122 (19.9)	62 (8.5)	184 (13.7)
	Continuous	492 (80.1)	665 (91.5)	1157 (86.3)
HIV status (*n* = 1341)	Reactive	90 (14.7)	144 (19.8)	234 (17.5)
	Non-reactive	524 (85.3)	583 (80.2)	1107 (82.6)
ART status (*n* = 234)	On ART	42 (48.3)	140 (95.2)	182 (77.8)
	Not on ART	45 (51.7)	7 (4.8)	52 (22.2)

*ART* anti-retroviral therapy, *CPT* cotrimoxazole prophylaxis therapy, *HIV* human immune virus, *TB* tuberculosis

With regard to TB patient loss to follow-up status by selected baseline socio demographic and clinical characteristics, majority of TB patient loss to follow-up (LTFU) occurred among males (83, 6.2%) and new TB patients at enrolment (106, 7.9%). In relation to type of health facility, majority of TB patient LTFU occurred among patients following treatment and care at health centers (Table 2).

**Table 2:** Tuberculosis patients’ loss to follow-up status by selected base line socio-demographic and clinical characteristics of 1341 TB patients enrolled into TB treatment and care at public health facilities, rural Ethiopia, during 2008–2015

Characteristics	Category	Follow-up outcome	
		LTFU *n* (%)	Censored *n* (%)
Age	Continuous	122 (9.1)	1219 (90.1)
Sex (*n* = 1341)	Male	83 (6.2)	704 (52.5)
	Female	39 (2.9)	505 (37.7)
Residence (*n* = 1341)	Urban	66 (4.9)	838 (62.5)
	Rural	56 (4.2)	380 (28.3)
Patient category (*n* = 1341)	New	106 (7.9)	1056 (78.7)
	Relapse	4 (0.3)	31 (2.3)
	Failure	0 (0)	3 (0.2)
	Transferred in	12 (0.9)	129 (9.6)
Distance (*n* = 1340)	≤10 km	73 (5.4)	1071 (79.9)
	> 10 km	49 (3.7)	147 (11.0)
Drug intensive (*n* = 1341	RHZE	120 (8.9)	1210 (90.3)
	RHZ	2 (0.1)	9 (0.7)
Drug continuous (*n* = 1322)	EH	47 (3.6)	410 (31.0)
	EH and RH	2 (0.2)	33 (2.4)
	RH	65 (4.9)	765 (57.9)
CPT provision at enrolment (*n* = 230)	Yes	19 (8.3)	182 (79.1)
	No	6 (2.6)	23 (10.0)
TB type (*n* = 1341)	P/pose	47 (3.5)	416 (31.0)
	P/negative	51 (3.8)	459 (34.2)
	EP	24 (1.8)	344 (25.7)
Treatment phase (*n* = 1341)	Intensive	85 (6.3)	99 (7.4)
	Continuous	41 (3.1)	1116 (83.2)
HIV sero status (*n* = 1341)	Reactive	36 (2.7)	196 (14.6)
	Non-reactive	85 (6.3)	1022 (76.2)
ART linkage status (*n* = 234)	On ART	12 (5.1)	170 (72.6)
	Not on ART	19 (8.1)	33 (14.1)
Type of health facility (*n* = 1341)	Health center	84 (6.3)	530 (39.5)
	Hospital	38 (2.8)	689 (51.4)

*ART* anti-retroviral therapy, *CPT* cotrimoxazole prophylaxis therapy, *HIV* human immune virus, *TB* tuberculosis, *RHZE* rifampicin + isoniazid + pyrazinamide + ethambutol, *EH* ethambutol

### Survival status and incidence of loss to follow-up

A total of 1341 patients were followed for a total of 7056.8 person months with median follow-up 5.3 months in TB treatment and care from 2008 to 2015. From this, tuberculosis patients were followed for a total of 3074.7 and 3974 person months of observations at health centers and general hospital, respectively. At the end of the observation period, of the total TB cases registered to TB treatment and care at heath facilities, 154(25.1%) were cured, 299 (48.7%) completed treatment, 24 (3.9%) died, three (0.5%) failed after treatment, 84(13.7%) were loss to follow-ups, and 50 (8.1%) were transferred into a health facility. Similarly, from the total TB cases registered to TB treatment and care at general hospital, 181 (24.9%) were cured, 441 (60.7%) completed treatment, 13 (1.8%) died, 38 (5.2%) were loss to follow-ups, and 54 (7.4%) were transferred into health facility.

**Fig. 1:**
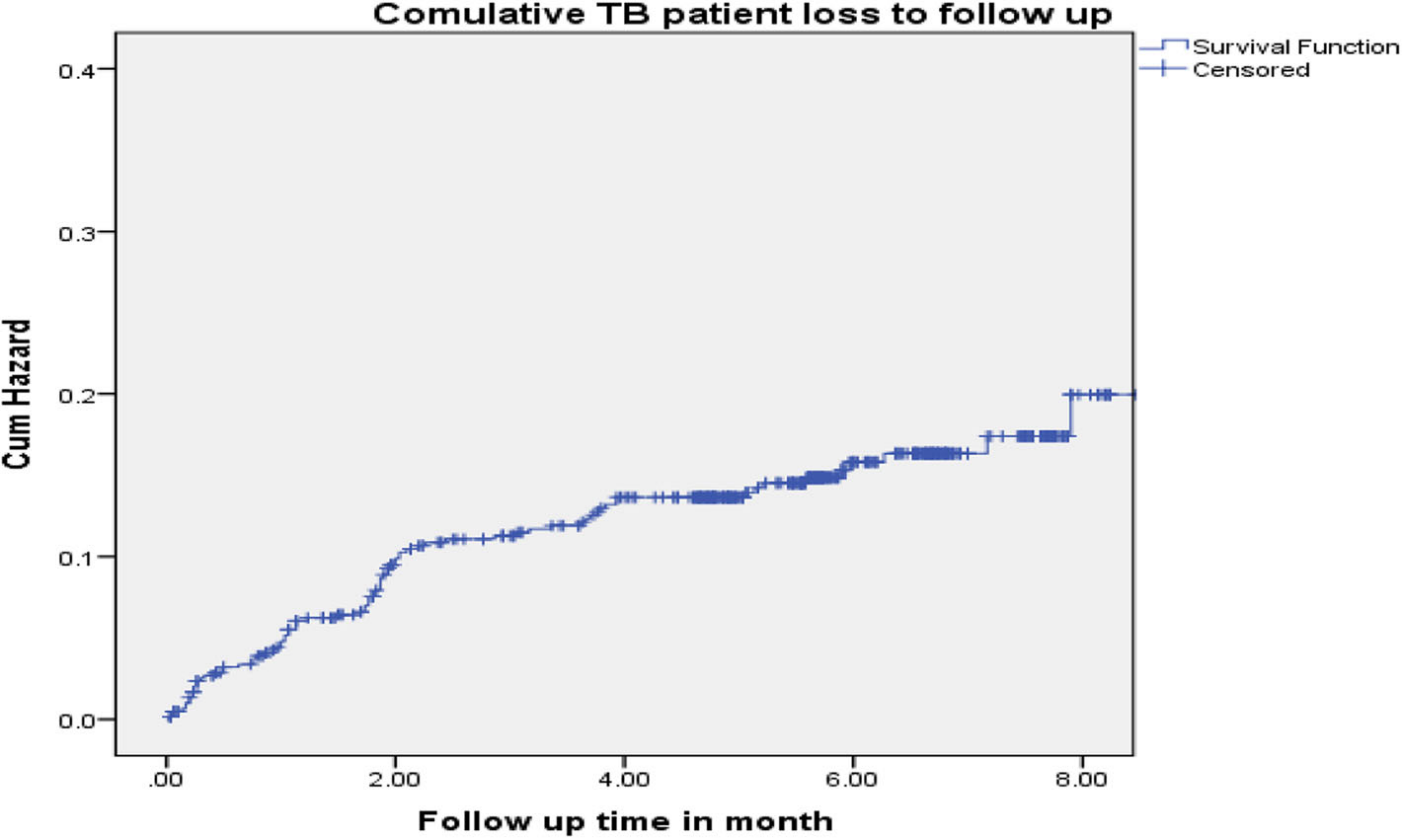
The TB patient loss to follow-up at the end of intensive and continuation period among tuberculosis patients registered to tuberculosis treatment and care at public health centers, rural Ethiopia, during 2008–2015

**Fig. 2:**
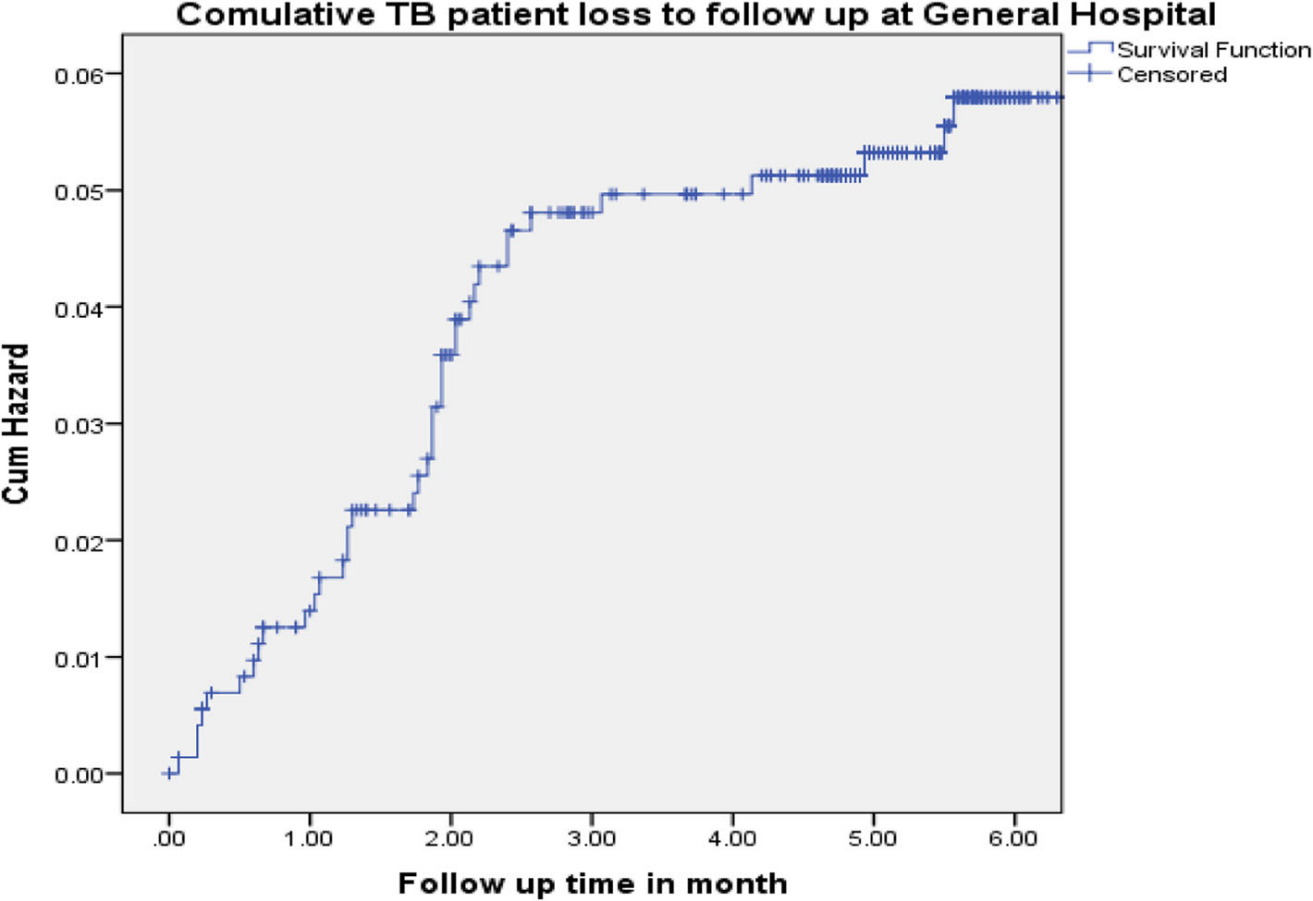
The TB patient loss to follow-up at the end of intensive and continuation period among tuberculosis patients registered to tuberculosis treatment and care at general hospital, rural Ethiopia, during 2008–2015

**Fig. 3:**
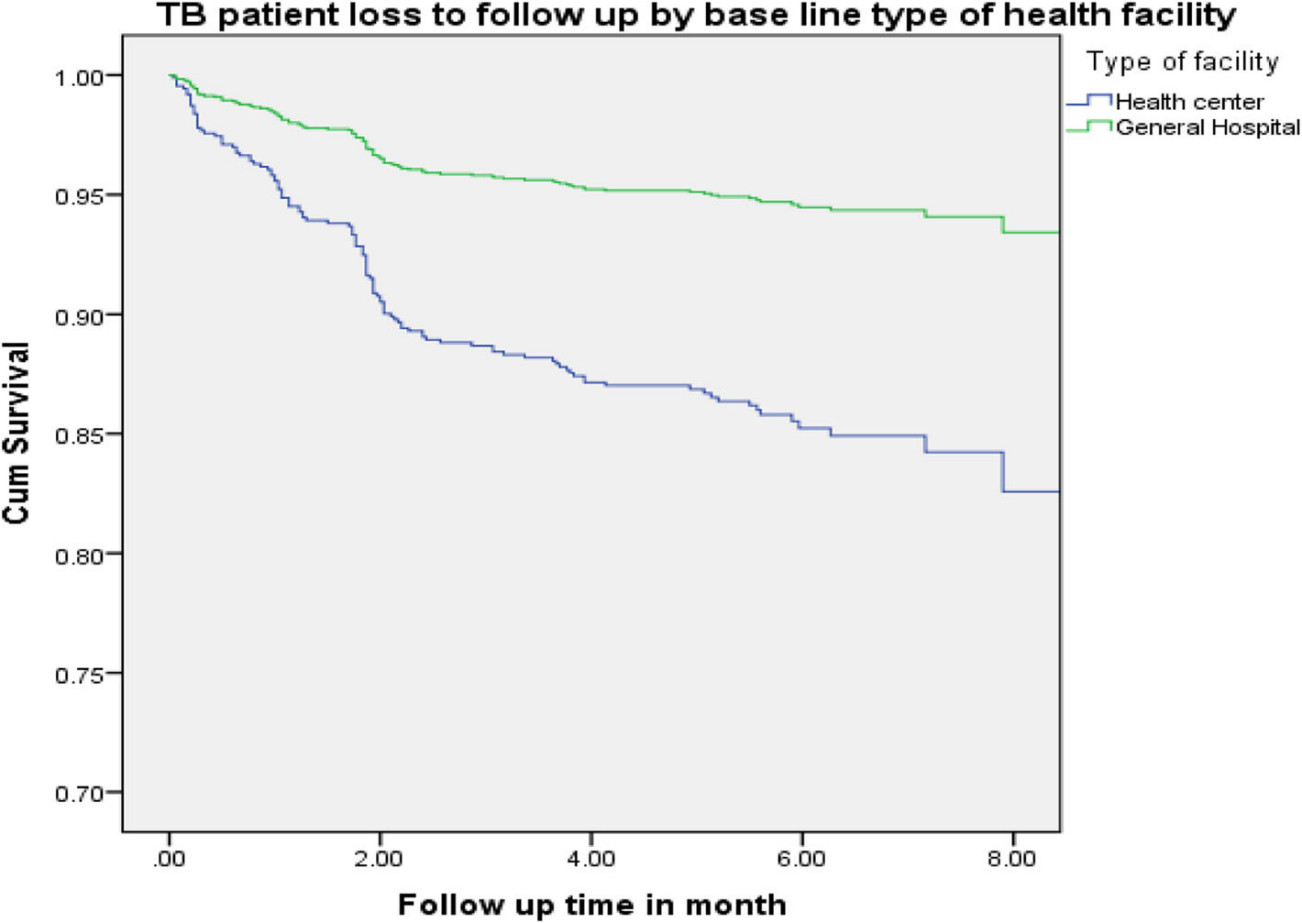
Survival experiences of loss to follow-up TB patients separated by baseline type of health facility among tuberculosis patients registered to tuberculosis treatment and care at health facilities, rural Ethiopia, during 2008–2015

The incidence of tuberculosis patient LTFUs rate on the cohort was 27.3 per 1000 person months and 9.6 per 1000 person months (PMs), at health centers and general hospital, respectively. From the overall loss to follow-ups that occurred, 55 (65.5%) and 33 (86.8%) of LTFUs occurred during the intensive phase and grew to 78 (92.9%) and 38 (100%) at health center and general hospital, respectively, at the end of 6-month observation period (Figs. 1 and 2). The life table analysis for survival function of TB patients follow-up between general hospital and health centers also revealed that the cumulative proportion surviving at the end of the intensive phase was 87% at health center whereas it was 95% for patients followed at the general hospital (Table 3). The median estimate of loss to follow-up time for TB patients who were attending treatment and care in hospital was 6.1 (95%CI, 5.9–7.3) months whereas for TB cases who were attending follow-up and care in health centers was 5.6 (95%CI, 5.3–5.8) months. According to the log rank test, the observed survival time differences between these two categories of patients receiving treatment and care at health centers and general hospital was statistically significant (*P* < 0.001) (Fig. 3).

**Table 3:** Life table for tuberculosis patients’ loss to follow-up status by type of health facility, for 1341 TB patients enrolled into TB treatment and care at public health facilities, rural Ethiopia, during 2008–2015

First-order Controls		Interval start time	Number entering interval	Number exposed to risk	LTFU	Proportion surviving	Cumulative proportion surviving at end of interval	Hazard rate
Type of facility	Health center	0.0	614	586	53	0.91	0.91	0.05
		2.0	505	488	20	0.96	0.87	0.02
		4.0	452	331	6	0.98	0.86	0.01
		6.0	204	121	3	0.98	0.84	0.01
		8.0	35	18.5	2	0.89	0.74	0.00
	General Hospital	0.0	727	709.5	25	0.96	0.96	0.02
		2.0	667	649	9	0.99	0.95	0.01
		4.0	622	438	4	0.99	0.94	0.00
		6.0	250	140	0	1.00	0.94	0.00
		8.0	30	15	0	1.00	0.94	0.00

Equally, the median estimate of survival time for TB patients who reside within the catchment distance of less than or equal to 10 km was 5.3 (95% CI, 5.1–5.5) months whereas for patients who come from distant areas was 4.6 (95% CI, 4.3–4.9) months. The difference in survival time between the two different categories of patients with regard to their distance from the nearest health facility was also statistically significant (*P* < 0.001).

At enrolment, the cumulative proportions of retention for TB patients were higher for patients who lived within the 10 km from the nearest public health facilities, who were HIV sero status negative and who were rural residents when compared to their counter parts.

### Independent predictors of loss to follow-up for TB patients

During bivariate analysis, age, sex, distance from the public health facility, residence, HIV sero status, and type of health facility were candidate variables for multiple cox model analysis of time to loss to follow-up of patients at health centers during TB treatment with a *p* value of < 0.25. Similarly, the age of the patient, sex, TB type, and HIV sero status were candidate variables during analysis of time to loss to follow-up of tuberculosis patients at general hospital.

During multiple Cox regression analysis, five variables were found to be significantly associated with time to loss to follow-up of TB patients. Variables significantly associated with time to loss to follow-up of TB patients included older ages (AOR = 1.7, 95%CI = [1.2–2.5], *P* < 0.001), being rural resident (AOR = 3.2, 95%CI = [1.9–5.3], *P* < 0.001), distance from the public health facility (AOR = 2.5, 95%CI = [1.5–4.2], *P* < 0.001), HIV sero status positive (AOR = 2.6, 95%CI = [1.6–4.2], *P* < 0.001), and following treatment and care at health center (AOR = 3.4, 95%CI = [2.1–5.5], *P* < 0.001) (Table 4).

**Table 4:** Bivariate and multivariable cox regression hazard model at public health facilities, rural Ethiopia, during 2008–2015

Characteristics	Category	CHR	95%CI	*P* value	AHR	95.0%CI AHR
Age in years	Continuous	2.1	1.5–3.0	*P* < 0.001	1.7	1.2–2.5
Sex						
	Male	1.5	1.0–2.3	0.065	1.5	0.98–2.17
	*Female	1			1	
Distance						
	*≤ 10 km	1			1	
	> 10 km	4.24	2.95–6.10	0.000	3.45	2.1–5.7
Residence						
	*Urban	1			**1**	
	Rural	1.9	1.3–2.7	0.000	2.7	1.6–4.6
HIV sero status						
	Reactive	2.2	1.5–3.2	0.000	2.2	1.5–3.2
	*Non-reactive	1			1	
Type of facility						
	Health center	2.8	1.92–4.13	0.000	3.38	2.1–5.5
	*Hospital	1			1	

*Reference category; *ART* anti-retroviral therapy, *CHR* crude odds ratio, *AHR* adjusted hazard ratio, *CI* confidence interval, *TB* tuberculosis

## Discussion

To attain WHO’s End TB strategy by 2030, TB patient treatment adherence is highly emphasized [2]. Thus, this study we aimed to determine the difference in time to LTFU of TB patients receiving treatment and care between health centers and general hospital, in rural Ethiopia. Accordingly, the incidence of tuberculosis patient loss to follow-up rate was 27.3 per 1000 PMOs and 9.6 per 1000 PMOs at health centers and general hospital, respectively. The time to loss to follow-up was significantly different between patients who were enrolled at health centers and general hospital. Although we did not get studies that compare the time to LTFU difference between TB patients receiving treatment and care at health centers and hospitals, we suggest that the significant time to LTFU difference might be explained from the fact that TB patient treatment and care service is more comprehensive in hospitals compared to health centers in Ethiopia [24, 26]. Thus, TB patients receiving treatment and care at facilities with low level of comprehensiveness might be forced to LTFU.

The incidence of TB patient LTFU recorded at health centers was lower when compared to finding from Morocco [28]. However, it was higher when compared to studies conducted across different parts of the world which includes Ethiopia [1], Ukraine [29], Ethiopia [30, 31], Dangila, Ethiopia [32], Cameroon [33], and South Africa [34]. The explanation for the variation could be explained from study setting and sample size. Unlike our study, which was conducted in rural and most peripheral part of Ethiopia, most studies mentioned in this comparison were conducted in areas adjacent to the main cities and their sample sizes were also much higher.

From the overall loss to follow-ups that occurred, 55 (65.5%) and 33 (86.8%) of LTFUs occurred during the intensive phase and grew to 78 (92.9%) and 38 (100%) at health center and general hospital respectively, at the end of 6-month observation period. This finding is greater compared to other studies, by 16% at the end of 6 months [1], by 13% (30% during intensive, 20% during continuous). This finding was lower in both intensive and continuous phase when compared to a study conducted in Pakistan [35] and Sudan [36]. This variation may be due to poor monitoring and tracking of loss to follow-up patients observed during early periods of treatment. The median estimate of loss to follow-up time for TB patients who were attending treatment and care in health centers was 5.6 (95%CI, 5.3–5.8) months, whereas for TB cases who were attending follow-up and care in hospital was 6.13 (95%CI, 5.9–7.3) months. The median time from treatment initiation to onset of loss to follow-up was short at health center compared to general hospital. This could be due to the delayed presentation and diagnosis of TB cases that lead to advancement of the disease, or it might be due to drug intolerance that resulted in early TB loss to follow-up. This finding is consistent with study in Thailand [37].

Over two thirds of TB/HIV co-infected patients were lost from care compared to TB patients whose HIV sero status was negative. The time to loss to follow-up for TB/HIV co-infected patients was nearly three-fold higher than those patients who were HIV sero status negative. Furthermore, the HIV non-reactive patients had much reduction in risk of loss to follow-up and improved survival time compared to those who were reactive for HIV, and the difference was statistically significant. This finding is consistent with other similar studies in that co-infected patients were more likely to loss to follow-up TB treatment relative to those whose HIV sero status was negative [38, 39]. Encouragingly, in TB–HIV co-infected patients, the receipt of ART was found to be protective against treatment loss to follow-up [40–42]. This could confirm the fact that TB is the most common opportunistic disease and the most common cause of burden in patients with HIV/AIDS infection in developing countries. But the mechanism by which the co-infection induces loss to follow-up from anti-TB treatment remains unclear [38].

In this study, TB patients with older age experienced loss to follow-up when compared to TB patients in younger age. This finding is consistent with a finding from Benin [38]. This could be plausible as this category of patients requests more social support from their communities. In addition, the duration of treatment, which is 6 to 8 months, is long in terms of separation from patients’ home towns and daily habits. Therefore, programs of DOTS at home or inside the community of origin could be a better TB surveillance strategy for the elder TB patients [38].

In this study, patients who lived > 10 km from the center had higher risk of early loss to follow-up when compared to those who lived within 10 km. This study is in line with finding from Uganda and Ethiopia [43, 44]. It was observed that the risk of non-adherence to treatment was higher among those who had lived > 10 km from the facility due to economic constrains to back transportation costs. Similarly a study from Philippines indicated that patients who lived near the service area were retained more on care as compared to those living abroad [44].

In this study, rural residence showed higher risk of TB patient loss to follow-up compared to their counter parts. This finding was consistent compared to other studies conducted in different settings. This may be explained by the fact that patients living in the rural area have little access to treatment centers in Ethiopia. Otherwise, they have to pay for public transport to reach treatment center, which could not be always possible as people in rural area have lower economic income.

Unlike other studies conducted so far, none of the variables did show significant association with time to patient loss to follow-up at general hospital in this study. Part of explanation for absence of significant association might be relatively small sample size as compared to other studies conducted on TB patient attrition at hospital level.

As part of limitation, the number of loss to follow-up might be overestimated or underestimated by exclusion of patients with undocumented outcomes. In addition, undetermined treatment outcomes of transfer out patients might have introduced bias too.

## Conclusions

Time to loss to follow-up from treatment and care between health centers and hospitals was significant. Significant TB patient loss to follow-up occurred in the first 2 months of TB treatment. In addition, there was a substantially higher retention probability in TB patients that were sero–negative for HIV, being an urban resident and living near to the service area. Strengthening the DOTs program especially during the intensive phase of treatment and tracking of loss to follow-ups is highly recommended. Moreover, TB patients receiving treatment and care in health centers deserve special attention.

## Abbreviations

ART: Anti-retroviral therapy
CPT: Cotrimoxazole prophylactic therapy
HIV: Human immunodeficiency virus
LTFU: Loss to follow-up
PMOs: Person months of observation
SSA: Sub-Saharan Africa
TB: Tuberculosis
